# The Health Impact of mHealth Interventions in India: Systematic Review and Meta-Analysis

**DOI:** 10.2196/50927

**Published:** 2023-09-04

**Authors:** Vibha Joshi, Nitin Kumar Joshi, Pankaj Bhardwaj, Kuldeep Singh, Deepika Ojha, Yogesh Kumar Jain

**Affiliations:** 1 Resource Centre Health Technology Assessment All India Institute of Medical Sciences Jodhpur Jodhpur India; 2 School of Public Health All India Institute of Medical Sciences Jodhpur Jodhpur India; 3 Department of Community Medicine and Family Medicine All India Institute of Medical Sciences Jodhpur Jodhpur India

**Keywords:** mobile applications, mobile apps, cost-benefit analysis, telemedicine, technology, India, patient satisfaction, pregnancy

## Abstract

**Background:**

Considerable use of mobile health (mHealth) interventions has been seen, and these interventions have beneficial effects on health and health service delivery processes, especially in resource-limited settings. Various functionalities of mobile phones offer a range of opportunities for mHealth interventions.

**Objective:**

This review aims to assess the health impact of mHealth interventions in India.

**Methods:**

This systematic review and meta-analysis was conducted in accordance with the PRISMA (Preferred Reporting Items for Systematic Reviews and Meta-Analyses) guidelines. Studies conducted in India, and published between April 1, 2011, and March 31, 2021, were considered. A literature search was conducted using a combination of MeSH (Medical Subject Headings) terms in different databases to identify peer-reviewed publications. Thirteen out of 1350 articles were included for the final review. Risk of bias was assessed using the Risk of Bias 2 tool for RCTs and Risk Of Bias In Non-randomised Studies - of Interventions tool (for nonrandomized trials), and a meta-analysis was performed using RevMan for 3 comparable studies on maternal, neonatal, and child health.

**Results:**

The meta-analysis showed improved usage of maternal and child health services including iron–folic acid supplementation (odds ratio [OR] 14.30, 95% CI 6.65-30.75), administration of both doses of the tetanus toxoid (OR 2.47, 95% CI 0.22-27.37), and attending 4 or more antenatal check-ups (OR 1.82, 95% CI 0.65-5.09). Meta-analysis for studies concerning economic evaluation and chronic diseases could not be performed due to heterogeneity. However, a positive economic impact was observed from a societal perspective (ReMiND [reducing maternal and newborn deaths] and ImTeCHO [Innovative Mobile Technology for Community Health Operation] interventions), and chronic disease interventions showed a positive impact on clinical outcomes, patient and provider satisfaction, app usage, and improvement in health behaviors.

**Conclusions:**

This review provides a comprehensive overview of mHealth technology in all health sectors in India, analyzing both health and health care usage indicators for interventions focused on maternal and child health and chronic diseases.

**Trial Registration:**

PROSPERO 2021 CRD42021235315; https://tinyurl.com/yh4tp2j7

## Introduction

The use of mobile computing and communication technologies in health care and public health are seen as a rapidly expanding area within eHealth. The World Health Organization’s Global Observatory for eHealth defined mobile health (mHealth) as “medical and public health practice supported by mobile devices, like mobile phones, patient monitoring devices, personal digital assistants, and other wireless devices” [1]. Devices used in mHealth interventions include laptops, tablets, mobile phones, smartphones, palmtops, notebooks, and netbooks.

Features of mobile technology, including mobility, instantaneous access, and direct communication, permit faster transfer of health information, which aid in medical and public health practices. mHealth services range from simple apps to complex technologies including voice messaging, SMS text messaging, multimedia message service, Bluetooth technology, and others, which could transform the worldwide delivery of health services, especially in low- and middle-income countries [1].

Various functionalities such as SMS text messaging, voice messaging, mobile internet browsing, Voice over Internet Protocol services (eg, Skype), instant messaging services, photographic capabilities, and a wide variety of device-based applications available through mobile technology offer a range of opportunities for mHealth interventions, such as text message and interactive voice response campaigns and content to mobile phone–based imaging (which have potential diagnostic capabilities) [2,3]. This technology has a broad extent and accessibility, which can be efficiently leveraged for health care delivery in areas where access is a major constraint [4].

mHealth is increasingly being used for medical services and public health practice for patient communication, monitoring, and education [5,6]. The interventions have also shown to reduce the burden of diseases linked with poverty and an improvement in the accessibility of the health services in terms of clinical diagnosis, treatment adherence, and chronic disease management [1,7-9]. There is considerable interest in mHealth interventions with an enormous potential for beneficial effects on health and health service delivery processes, especially in resource-limited settings such as India [10].

This paper provides a review of evidence regarding the health impacts of mHealth interventions in India. The purpose of this review is to assess health impact in terms of measurable changes in mortality, morbidity, disability-adjusted life years (DALYs), and improved disease detection rates.

## Methods

### Study Design

This systematic review and meta-analysis was conducted in accordance with the PRISMA (Preferred Reporting Items for Systematic Review and Meta-Analyses) guidelines [11]. Randomized controlled trials (RCTs), non-RCTs (including cluster RCTs and quasi-experimental studies), and prospective parallel cohort studies conducted in India were included. Studies published between April 1, 2011, and March 31, 2021, were considered, and the search was initiated on September 10, 2020, until March 10, 2021. Studies reported in the English language and conducted in India, which addressed the impact of mobile technology, using SMS text messaging or cellular telephone interventions for any disease (eg, diabetes, hypertension, cardiovascular disease, chronic respiratory disease, and cancer) and maternal and child health, and measured outcomes including morbidity, mortality, hospitalization rates, behavioral or lifestyle changes, the process of care improvements, clinical outcomes, patient and provider satisfaction, compliance, and cost-effectiveness, were included in the review.

### Literature Search

A literature search was conducted using a combination of text and Medical Subject Headings (MeSH) keywords in major databases, including PubMed, MEDLINE, Scopus, Cochrane Library, Web of Science, and Google scholar, to identify peer-reviewed publications. The MeSH keywords included the following: *Text Messaging*, *Health Literacy*, *Mobile Applications*, *Smartphone*, *Cell phone*, *Health Impact Assessment*, *Developing Countries*, *Multimedia*, *Cell Phone*, *Telemedicine*, *Medication Adherence*, *India*, *Hypertension*, *Primary Health Care*, *Risk Reduction Behavior*, *healthcare cost*, *Health Information Management*, and *Information Systems*. The search field was limited to the title or abstract (or both), and the type of publication was limited to original articles or full-length research articles. We excluded cross-sectional studies, letters, case reports, study protocols, reviews, opinions, gray literature, and non–peer-reviewed publications. The reference lists of articles were also examined to identify other potentially relevant articles. The protocol for this systematic review and meta-analysis has been registered in PROSPERO 2021 (CRD42021235315).

### Study Selection and Characteristics

Two researchers (VJ and DO) independently screened the titles and abstracts to identify potentially eligible studies, and further assessment was performed by 2 authors (NKJ and YKJ). Only full-text articles published between 2011 and 2021, written in the English language, were included. The authors excluded duplicates and studies conducted outside India.

**Figure 1:**
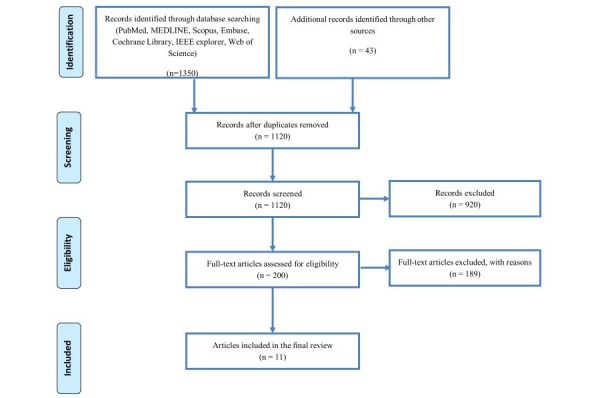
PRISMA (Preferred Reporting Items for Systematic Reviews and Meta-Analyses) flow diagram for database searches of studies on mobile health interventions conducted in India in 2011-2020.

Initial searches identified 1393 titles. After removing duplicates, 1120 articles were included for initial screening. Of these, 920 articles were excluded after screening by title and abstract, leaving 200 articles, which were considered in more detail. A further 187 papers were subsequently excluded for not meeting the relevant criteria. Thirteen of the eligible studies were intervention studies, comprising 3 RCTs; 5 quasi–RCTs; 1 cluster RCT; 1 prospective, parallel-group cohort study; and 1 quantitative, single-arm, pretest, posttest interventional study (Figure 1).

### Data Extraction

The extracted data included the names of the authors, year of publication, study design, study location, sampling, and main results. All these details were captured and recorded in an Excel (Microsoft Corp) spreadsheet. The information reported in or calculated from the included studies was used for analysis. Corresponding authors of the articles were not contacted for unpublished or additional information. Disagreements related to the inclusion of an article were resolved through consensus among the authors.

### Quality Assessment and Assessment of Risk of Bias

Risk of bias of each study was assessed using the Risk of Bias 2 tool for RCTs and Risk Of Bias In Non-randomised Studies - of Interventions for non-RCTs [12,13]. Risk-of-bias grading for the different components of each study is shown in Table 1. Four of the intervention studies were graded as being at low risk of bias, 6 as moderate, and 1 as high.

**Table 1:** Characteristics and results of studies investigating the effectiveness of mobile health (mHealth) interventions in India during 2011-2020.

Study (year; location) [overall risk of bias]	Study population	Intervention and control	Results	Study outcome
Prinja et al [14] (2017; Uttar Pradesh, India) [low]	Population: data obtained from the 2011 AHS^a^ and 2015 CEAHH^b^ survey among women or mothers with 1-year-old children Preintervention: 1508 ASHAs^c^ (intervention: n=99; control: n=99); postintervention: 1028 (intervention: n=534; control: n=534)	Intervention: pregnant women and mothers using an mHealth app; control: women and mothers not using mHealth applications	Increase in the coverage IFA^d^ supplementation (12.58%; 95% CI 0.086-0.27) Self-reporting of illnesses or complication during pregnancy (13.11%) and after delivery (19.6%) The coverage of ≥3 ANC^e^ visits (10.3%; 95% CI 0.039-0.98) Coverage of ≥2 tetanus toxoids (4.28%; 95% CI 0.055-0.68) Institutional delivery (95% CI 0.044-0.59) Full immunization (95% CI 0.20-1.032) No change in the quality of ANC care	Significant improvement in IFA supplementation, identification, and self-reporting of illnesses during pregnancy and after delivery
Modi et al [15] (Gujarat, India) [low]	Population: rural tribal communities of Gujarat, India (neonates and mothers); population: 22 PHC^f^ clusters (intervention: n=11; control: n=11)	Intervention (with an mHealth package): 11 PHCs and 280 ASHAs; population: 234,134 Control (without an mHealth package): 11 PHCs and 281 ASHAs; population: 242,809	ANC of ≥4: intervention (n=622, 79.2%); 89.5, 95% 87.6-91.3); control (88.7, 95% CI 86.6-90.6) TT^g^ during the last pregnancy: intervention (n=771, 98.2%; 98.2, 95% CI 97.4-98.9); control (n=694, 98.3%; 96.8, 95% CI 96-97.6) Delivered at an institution or hospital: intervention (n=580, 73.9%; 83.2, 95% CI 80.4-85.9); control (n=600, 85.0%; 84.9, 95% CI 82.1-87.6) ASHAs present during delivery: intervention (n=267, 34.0%); control (n=267, 37.8%) MACCI^h^: intervention (31%); control (31%) ASHA visit at home at least twice in the first week of delivery: intervention (n=149, 19.0%; 32.4, 95% CI 29.7-35.1); control (n=99, 14.0%; 22.9, 95% CI 20.2-25.6) Low Birth Weight (≤2 kg) at the time of birth: intervention (3.5, 95% CI 2.3-4.7); control (6.6; 95% CI 5.4-7.8) Practice breastfeeding at 6 months: intervention (n=151, 19.2%; 57.4, 95% CI 54.1-60.8); control (n=95, 13.5%; 45.1, 95% CI 41.8-48.4)	ImTeCHO^i^ mobile apps and web-based applications, ASHAs, and PHC staff improved the coverage and quality of MNCH^j^ services in difficult-to-reach areas Improvement in coverage home visits by ASHAs during the antenatal period, postnatal period, early initiation of breastfeeding, and exclusive breastfeeding
Murthy et al [16] (Mumbai, India) [moderate]	2016 pregnant women, aged 18 years or older: intervention (n=500); control (n=1516); analyzed (intervention: n=1038; control: n=379); time 1 (intervention: n=1516; control: n=500); time 2 (intervention: n=1113; control: n=402); time 3 (intervention: n=1038; control: n=379)	Intervention group received mMitra voice messages twice per week throughout their pregnancy and until their infant turned 1 year of age Control group received no mMitra voice message	Infant care practices that the intervention group performed better: infant feeding at 6 months of age (OR^k^ 1.4, 95% CI 1.08-1.82; *P*=.009), fully immunizing the infant (OR 1.531, 95% CI 1.141-2.055; *P*=.005) Control group performed better on practices: increase in baby weight within 3 months (*P*=.03; OR 0.77, 95% CI 0.6-0.98) In infant care knowledge: increase in baby solid food by 6 months in the intervention group (OR 1.89, 95% CI 1.371-2.605; *P*<.01); the ideal birth weight is >2.5 kg (OR 2.279, 95% CI 1.617-3.213; *P*<.01)	mMitra voice-based mHealth intervention to demonstrate a positive impact on infant birth weight—a health outcome of public health importance
Ilozumba et al [17] (2018; Jharkhand, India) [low]	Population: women between the ages of 18 and 45 years who had delivered a baby in the past 1 year (N=2200; intervention: n=733; control: n=739)	The study has 3 groups, all of which received standard care government programs that included the recruitment and support of ASHAs: An intervention group that received MfM^l^ in addition to an NGO’s^m^ existing interventions A quasi-control group that received NGO programs A standard care group that only received standard care government programs	The odds of having a higher score on maternal health knowledge significantly increased when comparing intervention and control groups Women in the MfM group were more likely to attend 4 or more ANC visits than those in the standard care group (OR 1.36, 95% CI 1.30-1.42) and the NGO group (OR 1.23, 95% CI 1.17-1.29) The odds of a women in the MfM group were significantly higher than the odds of women in the standard care group (OR 1.34, 95% CI 1.28-1.41) and the NGO group (OR 1.19, 95% CI 1.13-1.25) Higher maternal health knowledge -MfM versus standard care (intervention: OR 1.19, 95% CI 1.13-1.25; control [reference] OR 1.00) Attended 4 or more ANC visits (intervention: OR 1.38, 95% CI 1.32-1.44; control [reference] OR 1.00) Delivered at a health facility (intervention OR 1.35, 95% CI 1.29-1.42)	This study showed that women in the intervention group reported higher levels of maternal health knowledge than those in the NGO intervention or those who received standard care The primary outcomes of interest were maternal health knowledge, ANC attendance, and delivery in a health facility
Prinja et al [18] (2018; Uttar Pradesh, India) [low]	Population: data obtained from the 2011 AHS and 2015 CEAHH survey among women or mothers with 1-year-old children Preintervention: 1508 ASHAs (intervention: n=99; control: n=99); postintervention: 1028 (intervention: n=534; control: n=534)	Intervention: pregnant women and mothers using an mHealth app; control: women and mothers not using mHealth applications	ReMiND^n^ resulted in a cost saving of US $90 per DALY^o^ averted US $2569 per death averted. From the health system perspective, ReMiND incurred an incremental cost of ₹12,993 (US $205) per DALY averted and ₹371,577 (US $5865) per death averted	mHealth intervention as part of the ReMiND program is cost-saving from a societal perspective
Modi et al [19] (2020; Gujarat, India) [low]	Population: rural tribal communities of Gujarat, India (neonates and mothers; population: N=22 PHC clusters: intervention: n=11; control: n=11)	Intervention (with an mHealth package): 11 PHCs and 280 ASHAs; population: n=234,134 Control (without an mHealth package): 11 PHCs and 281 ASHAs; population: n=242,809)	ImTeCHO is a cost-effective intervention at an incremental cost of US $74 per life years saved or US $5057 per death averted Total births in the study area (n=3014) Cost per live birth (US $54) Cost per 1000 live births (US $54,360) Infant deaths averted per 1000 live births (n=11) Life years saved (life expectancy=68.35 years; n=735) Cost per infant deaths averted (US $5057) Cost per life years saved due to infant deaths averted (US $74) IMR^p^ as intention-to-treat in the study area (cost per ASHA (US $578.95)	mHealth intervention as part of the ImTeCHO program is cost-effective and should be considered for replication
Pfammatter et al [20] (2015; India) [moderate]	Population: adults aged 18 years and older (N=1925; intervention: n=611; control: n=632)	Intervention: 1 million Nokia subscribers who opted into mDiabetes for 6 months Control: non-Nokia phone subscribers	Intervention group: 24.71% of them improved their fruit and vegetable intake and reduced their fat intake; 128 (20.95%) improved their preventive behavior Control group: 36.55% decline in the number of participants’ healthy behaviors; 73 (11.55%) improved their preventive behavior	A text messaging intervention was feasible and showed initial evidence of effectiveness in improving diabetes-related health behaviors
Kleinman et al [21] (2017; India) [low]	Population: aged 18-65 years with type 2 diabetes 6 months from baseline (N=90; intervention: n=44; control: n=46)	Intervention: participants received the mHealth app and a mobile phone data stipend for 6 months Control: manage their diabetes as usual	Primary outcome: intervention mean 1.1-1.5; control mean 0.8-1.6 (*P*=.02) Secondary outcomes: intervention mean 32.6-66.4; control mean 23.5-70.0 (*P*=.55) BMI change: intervention mean 0.1-1.0; control mean 0.1-1.1 (*P*=.53)—patient-reported values improved from baseline to 6 months (intervention: n=16, 39.0%; control: n=5, 12.8%; *P*=.03) Medication adherence (intervention: 39.0%; control: 12.8%; *P*=.03) Increased frequency of blood glucose self-testing (intervention: 39.0%; control: 10.3%; *P*=.01)	Significantly more participants in the intervention group than in the control group
Prabhakaran et al [22] (2019; India) [moderate]	Population: rural population (CHCs^q^), ≥30 years of age, confirmed diagnosis of hypertension or diabetes mellitus Population: 40 clusters; intervention: n=20 (mWellcare) 20 clusters; 1842 participants enrolled (N=2140); control: 20 CHCs (allocated to EUC^r^) and 20 clusters; 1856 participants enrolled (N=2130)	Effectiveness of the mWellcare app for 5 chronic conditions (hypertension, diabetes mellitus, current tobacco and alcohol use, and depression) vs usual care (intervention group: [mWellcare arm]: EUC NCD^s^ nurses with the mWellcare system; control group: EUC NCD nurses Without the mWellcare system)	Primary outcomes: Change in SBP^t^: control: mean –12.7 mm Hg; intervention: mean –13.7 mm Hg (effect size –0.3, adjusted 95% CI –3.9 to 3.3; *P*=.87) Change in HbA1c^u^: control: mean –0.58%; intervention: –0.48% (effect size 0.08, adjusted 95% CI –0.27 to 0.44; *P*=.66) Secondary outcomes: Change in fasting blood glucose: control: mean –22.7 mg/dL; intervention: –15.0 mg/dL (effect size 8.4, adjusted 95% CI –9.6 to 26.5; *P*=.37) Change in total cholesterol: control: mean 2.0 mg/dL; intervention: mean 0.1 mg/dL (effect size –2.5, adjusted 95% CI –7.1 to 2.0; *P*=.29) Change in CVD^v^ risk score: control: mean 0.6%; intervention: 2.4% (effect size –0.4, adjusted 95% CI –2.3 to 1.5; *P*=.66) Change in BMI: control: mean 0.08 kg/m2; intervention: 0.16 kg/m2 (effect size –0.05, adjusted 95% CI –0.47 to 0.37; *P*=.82) Change in tobacco use: control: mean –7.0%; intervention: mean –0.6% (effect size –0.8, adjusted 95% CI –5.7 to 4.2; *P*=.76) Change in alcohol use: control: mean –3.8%; intervention: mean –2.4% (effect size 0.7, adjusted 95% CI –3.7 to 5.1; *P*=.74) Change in alcohol use score: control: mean 10.0; intervention: 9.4 (effect size –0.6, adjusted 95% CI –3.2 to 2.1; *P*=.68) Change in depression score: control: mean 12.4; intervention: mean 10.9 (effect size –1.6, adjusted 95% CI –4.4 to 1.2; *P*=.28)	Incremental benefit of mWellcare over enhanced usual care in chronic conditions The trial did not find any significant difference in the primary outcomes, that is, reduction in SBP or HbA1c, and Secondary outcomes, that is, fasting blood glucose, total cholesterol, predicted 10-year risk of CVD, BMI, depression, and tobacco and alcohol use between the 2 arms
Garner et al [23] (2020; India) [moderate]	Population: urban slum and rural slum dwellers (n=346) Pretest (n=87): those who earned an 8 or above on the pretest paired t test Posttest (n=259): those who earned a 7 or below on the pretest	Intervention through an mHealth app to improve hypertension health literacy	Study aim 1: to assess the effectiveness of an mHealth app to improve hypertension health literacy among participants in India Study aim 2: to estimate relationships between participant hypertension health literacy and sociodemographic variables Pretest: participants who performed moderately well on the pretest also had improved posttest scores (significant mean difference between pretest and posttest scores 2.49; *P*<.001 [paired t test])	The mHealth app provides an effective and valuable culturally tailored educational resource for nurses and other health to improve hypertension health literacy among populations in India
Gautham et al [24] (2015; Tamil Nadu, India) [high]	Population: rural health providers (n=16) and patients (n=126; experimental: n=65; control: n=61)	Intervention group: given applications on their mobile phones Control group: no application given; only the phone and a set of paper guidelines to use in the field	Control group scored significantly higher than the experimental group (control group: mean 13.68; experimental group: 9.51; *P*<.05) in the posttraining evaluation. Control: mean pretraining score 8.58 out of 19 (SD 2.03); experimental: mean pretraining score 7.01 out of 19 (SD 1.85; *P*=.19) Control: mean posttraining score 13.68 out of 19 (SD 2.17); experimental: mean posttraining score 9.51 out of 19 (SD 2.48; *P*<.05)	This study supports the implication that mMRIGs^w^ comprise a feasible and effective solution for standardizing and enhancing the quality of care delivered by millions of frontline rural health providers with varying levels of training and literacy
Praveen et al [25] (2014; Andhra Pradesh, India) [moderate]	Population: ASHAs, NPHWs^x^, and PHC physicians. 227 adults screened by ASHAs, 65 adults screened by PHC physicians	The CDSS^y^ was field-tested in 11 villages and 3 PHCs. CVD risk factor profile for participants screened by ASHAs (n=227) and doctors (n=65)	The CDSS recommend referral to a doctor to 128 of 227 adults and did not recommend referral to 99 of 227 adults. High CVD risk was noted in 88 of 128 (69%) adults, and in another 40 of 99 (31%) adults. Blood pressure lowering medication given to 29 of 65 (45%) adults and not to 36 of 65 (55%) adults. The other assessment of behavior change (COM-B^z^ model) revealed 3 themes: (1) potential to transform prevailing health care models, (2) task-shifting of CVD screening to the ASHA was the central driver of change, and (3) system-level barriers such as access to doctors and medicines are still present	A tablet-based CDSS implemented within primary health care systems has the potential to help improve CVD outcomes in India
Jadhav et al [26] (2016; Maharashtra, India) [moderate]	Population: adults aged 18-20 years having a personal mobile phone with SMS text messaging capability (N=400; control: n=200; intervention: n=200)	Intervention group: the message was reinforced through SMS text messages from mobile phones Control: no oral health–related SMS text messages or any kind of health education was given to the participants	Gender-wise distribution of participants: 137 male and 63 female participants in the intervention group and 149 male and 51 female participants in the control group (*P*>.05) Mean OHI^aa^ score at different intervals between the intervention and control groups showed no significant difference at baseline (*P*=.28) and after the first month (*P*=.58); however, it was significantly lower in the intervention group after the second, third, and sixth months (*P*<.01) Mean GI^ab^ scores at different intervals between the intervention and control groups were significantly no different at baseline (*P*=.39) and after the first month (*P*=.85); however, it was significantly lower in the intervention group after the second, third, and sixth months (*P*<.01)	Reinforcement of oral health education through SMS text messages is effective media to improve oral health

^a^AHS: Annual Health Survey.

^b^CEAHH: cost-effectiveness analysis household.

^c^ASHA: accredited social health activist.

^d^IFA: iron–folic acid.

^e^ANC: antenatal care.

^f^PHC: primary health center.

^g^TT: Tetanus toxoid.

^h^MACCI: modified accredited social health activist–centric composite coverage index.

^i^ImTeCHO: Innovative Mobile Technology for Community Health Operation.

^j^MNCH: maternal, neonatal, and child health.

^k^OR: odds ratio.

^l^MfM: Mobile for Mothers.

^m^NGO: nongovernmental organization.

^n^ReMiND: reducing maternal and newborn deaths.

^o^DALY: disability-adjusted life year.

^p^IMR: infant mortality rate.

^q^CHC: community health center.

^r^EUC: enhanced usual care.

^s^NCD: noncommunicable disease.

^t^SBP: systolic blood pressure.

^u^HbA_1c_: hemoglobin A_1c_.

^v^CVD: cardiovascular disease.

^w^mMRIG: media-rich interactive guideline.

^x^NPHW: nonphysician health care worker.

^y^CDSS: clinical decision support system.

^z^COM-B: capability, opportunity, and motivation.

^aa^OHI: Oral Hygiene Index.

^ab^GI: Gingival Index.

### Meta-Analysis

There was substantial heterogeneity among studies in their mHealth interventions and outcomes, except for studies on maternal, neonatal, and child health. Consequently, we performed a random-effects meta-analysis using the Mantel-Haenszel method in RevMan [27] for 3 comparable studies, which had all used cell phones rather than routine prenatal care as the intervention and had assessed increases in the number of antenatal check-ups, tetanus toxoids administered to pregnant women, institutional deliveries, and iron–folic acid to assess the effect of health care usage. However, as the relevant intervention for the purpose of this review, we exclusively compared the cell phone group to the usual care group in the meta-analysis. However, given the small number of studies, we did not undertake possible sensitivity analyses.

## Results

### Types of Outcomes Examined

Four studies examined the indicators of maternal, neonatal, and child health [14-17]—these reported the number of antenatal check-ups [14,15,17]; birth weight [15]; institutional delivery [14-17]; knowledge of the danger signs of pregnancy [14,15]; indicators of infant feeding and breastfeeding [14]; usage of antenatal, intrapartum, and postnatal care [14,15,17]; indicators of self-efficacy [15,17]; uptake of immunization [14,15]; and maternal health knowledge [17]. We found two studies evaluating the cost-effectiveness of mHealth programs [18,19]. Other outcomes included improvement in diabetes risk behaviors and increased awareness about the causes and complications of diabetes [20], improvement in medication adherence and the frequency of blood glucose testing [21], change in systolic blood pressure and hemoglobin A_1c_ levels [22], quality of care delivered by primary health workers [23-25], and oral health education [26]. The results are organized below in accordance with the types of outcomes examined in each study.

### Effects on Maternal, Neonatal, and Child Health

A pre-post quasi-experimental study used an mHealth application in the Kaushambi district in Uttar Pradesh, India, to increase the quality of counseling by community health volunteers, resulting in improved uptake of maternal, neonatal, and child health services. A significant increase in coverage iron–folic acid supplementation and identification and self-reporting of illnesses or complications during pregnancy and after delivery were seen in the intervention area, but there was no change in the quality of antenatal care (ANC) care [14]. Similarly, an mHealth application was used in an open cluster RCT conducted in 22 primary health centers in 6 tribal blocks of Bharuch and Narmada districts in Gujrat, India, to assess the increase in the coverage of maternal, neonatal, and child health services and that of at least 2 home visits by accredited social health activists within the first week of birth. There were significant improvements in coverage home visits by accredited social health activists during the antenatal and postnatal period, early initiation of breastfeeding, and exclusive breastfeeding [15].

A pseudo-RCT conducted in Mumbai (Maharashtra, India) by Murthy et al [16], assessed the impact of age- and stage-based mobile phone voice messaging for pregnant women on reduction in low birth weight and child malnutrition and improvement in women’s infant care knowledge and practices. They observed that the intervention group performed well in infant care practice indicators: administering supplementary feeding to the infant at 6 months of age (odds ratio [OR] 1.4, 95% CI 1.08-1.82; *P*=.009) and fully immunizing the infant (OR 1.531, 95% CI 1.141-2.055; *P*=.005). Moreover, women in the intervention group had increased knowledge of giving infants solid food by 6 months of age and of the fact that the ideal birth weight is >2.5 kg [16]. A study from Jharkhand used a mobile app to support home visits by community health workers; Ilozumba et al [17] found that women receiving the mHealth intervention were more likely to attend 4 or more ANC visits and had significantly higher odds of delivering a baby at a health center than those receiving standard care and those receiving other interventions from a nongovernmental organization. Moreover, the usage of ANC services and delivery at a health center were associated with the education level of the spouse [17].

### Cost-Effectiveness

Prinja et al [18] assessed the cost-effectiveness of the ReMiND (reducing maternal and newborn deaths) program in Uttar Pradesh, India; both the societal and health care perspectives were taken into account. Overall, the ReMiND program was considered a cost-saving intervention from the societal perspective. It resulted in a cost saving of US $90 per DALY averted US $2569 per death averted. From the health system perspective, the ReMiND program incurred an incremental cost of ₹12,993 (US $205) per DALY averted and ₹371,577 (US $5865) per death averted [18]. A study conducted in Gujrat, India, found the ImTeCHO (Innovative Mobile Technology for Community Health Operation) intervention to be cost-effective at an incremental cost of US $74 per life-years saved or US $5057 per death averted [19].

### Effect on Chronic Conditions

Study conducted by Pfammatter et al [20] to examine the effect of mDiabetes—a text messaging program to improve diabetes risk behaviors—on fruit, vegetable, and fat intake and exercise among Nokia phone users in India. A greater improvement in the health behavior composite score over 6 months was observed among participants who received the text messages than among those who did not receive text messages [20]. An RCT conducted by Kleinman et al [21] at 3 sites in India assessed the impact of an mHealth diabetes platform on clinical outcomes, patient-reported outcomes, patient and provider satisfaction, and app usage. There was decrease of 1.5% in mean hemoglobin A_1c_ levels in the intervention group and 0.8% in the usual care group, an improvement in self-reported medication adherence from baseline, and an increase in blood glucose testing in the intervention group from baseline compared to that in the control group (39.0% vs 10.3%, respectively; *P*=.01) [21]. Prabhakaran et al [22] conducted a cluster-RCT using the mWellcare system for integrated management of 5 chronic conditions (hypertension, diabetes mellitus, current tobacco and alcohol use, and depression). No evidence of difference in systolic blood pressure and hemoglobin A_1c_ levels was observed between the intervention and control groups [22].

### Other Effects

Garner et al [23] determined the effectiveness of an mHealth application to improve hypertension health literacy among vulnerable populations in India. A significant improvement in the understanding of hypertension through the innovative animated application was observed [23]. In the RCT conducted in rural areas of Tamil Nadu, India, Gautham et al [24] observed that mobile app–based procedural guidance for rural frontline health care providers had significant potential for attaining consistently standardized quality of care with patients’ acceptance. Praveen et al [25] showed that implementation of a mobile clinical decision support system for cardiovascular disease management by public nonphysician health care workers and physicians in a rural Indian setting increased the number of referrals to the physician and had potential to help improve cardiovascular disease outcomes, but system-level barriers have an impact on limiting the access to medical care. Jadhav et al [26] assessed the effectiveness of the reinforcement of oral health education SMS text messages and reported that mean Oral Hygiene Index and Gingival Index scores in the intervention group were significantly lower than those in the control group (*P*<.01).

### Effect on Health Care Usage

**Figure 2:**
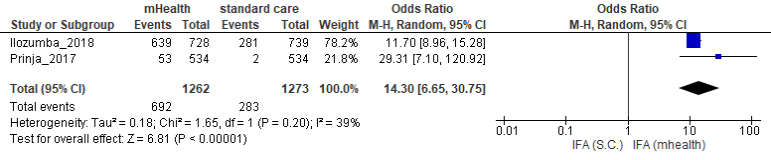
Meta-analysis of the effect of mobile health interventions versus standard care on the intake of complete doses of iron–folic acid supplements during prenatal care. IFA: iron–folic acid; mHealth: mobile health; OR: odds ratio; SC: standard care.

**Figure 3:**
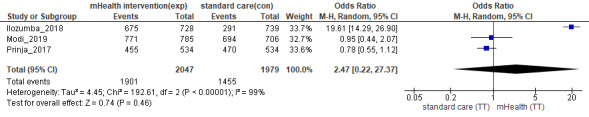
Meta-analysis of the effect of mobile health interventions versus standard care on taking 2 doses of the tetanus toxoid during pregnancy. mHealth: mobile health; OR: odds ratio; TT: tetanus toxoid.

**Figure 4:**
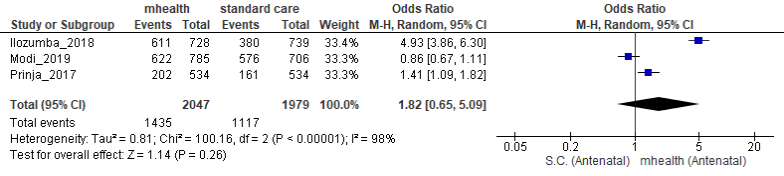
Meta-analysis of the effect of mobile health interventions versus standard care on 3 or more antenatal care check-ups conducted during pregnancy. mHealth: mobile health; OR: odds ratio; SC: standard care.

Among pregnant women, those using mHealth interventions were more likely to take a complete dose of iron–folic acid supplements (OR 14.30, 95% CI 6.65-30.75; Figure 2), both doses of the tetanus toxoid (OR 2.47, 95% CI 0.22-27.37; Figure 3), and to attended 4 or more antenatal care check-ups (OR 1.82, 95% CI 0.65-5.09; Figure 4) than those who received routine prenatal care. No strong evidence of differences regarding institutional deliveries (OR 1.14, 95% CI 0.26-4.95) were found.

## Discussion

### Principal Findings

mHealth is an implicit, promising tool for addressing several health care system limitations in transitional countries, such as a limited health care workforce, scarce resources, high burden of disease, rapid population growth, and challenges of extending health care to underserved populations. We identified 13 studies showing the impact of mobile technology–based interventions designed to improve health care service delivery processes in the Indian setting. Most studies were at moderate and low risk of bias. Heterogeneity among studies did not allow the calculation of a pooled estimate for all the parameters. However, a meta-analyses of 3 studies arbitrated to be sufficiently homogenous showed that mHealth interventions used for maternal and child health improved the usage of prenatal services including the intake of a complete dose of iron–folic acid supplements, taking both doses of the tetanus toxoid, and attending 4 or more antenatal care check-ups. No strong evidence of differences regarding institutional deliveries were found. A similar review conducted by Lee et al [28] for low- to middle-income countries showed that mHealth technologies are rapidly being used to promote health care use, improve the quality of pre- and postnatal care, and collect data on pregnancy and child health.

In our systematic review, we could not use economic evaluation–tailored reporting standards (such as the CHEERS [Consolidated Health Economic Evaluation Reporting Standards] checklist [29]) for full economic evaluation due to the lack of sufficient economic evaluation studies, as indicated by Iribarren et al [30], who described the evidence related to economic evaluations of mHealth interventions in low- to middle-income countries and in the evaluation of 2 mHealth interventions in India: ReMiND [18] and ImTeCHO [19]. These studies included a comparison of the effectiveness of a health-related outcome and reported economic data. Both the studies showed a positive economic impact considering the societal perspective.

All the studies included in this review provide evidence that the interventions conducted for the chronic diseases had an impact on clinical outcomes, patient and provider satisfaction, app usage, and improvement in health behavior (except for the study conducted by Prabhakaran et al [22]). Similar findings were described in the review conducted by Beratarrechea et al [31] for chronic diseases in transitional countries, which addressed more than 1 outcome and reported a positive impact on chronic disease outcomes.

### Limitations and Conclusion

This paper reviews the comprehensive use of mHealth technologies in all sectors of health care in India. We used a thorough, extensive, and highly sensitive literature search technique in this systematic review, which analyses both health and health care usage indicators, encompassing the entire scope of relevant mHealth technologies including those focusing on maternal and child health and chronic diseases. All comparative reviews have been conducted for low- to middle-income countries and mainly focused on the either chronic disease or maternal and child health [28,30-38].

However, due to a small number of studies for a single set of interventions, a meta-analysis for all the impact indicators was not conducted. Additional work is needed to improve and test this with a larger set of interventions, and to determine how to best integrate it with different conceptual frameworks that have been published.

## Appendix

Multimedia Appendix 1 PRISMA (Preferred Reporting Items for Systematic Reviews and Meta-Analyses) checklist.

## Abbreviations

ANC: antenatal care
CHEERS: Consolidated Health Economic Evaluation Reporting Standards
DALY: disability-adjusted life year
ImTeCHO: Innovative Mobile Technology for Community Health Operation
MeSH: Medical Subject Headings
mHealth: mobile health
OR: odds ratio
PRISMA: Preferred Reporting Items for Systematic Reviews and Meta-Analyses
RCT: randomized controlled trial
ReMiND: reducing maternal and newborn deaths
